# Jellyfish as Prey: Frequency of Predation and Selective Foraging of *Boops boops* (Vertebrata, Actinopterygii) on the Mauve Stinger *Pelagia noctiluca* (Cnidaria, Scyphozoa)

**DOI:** 10.1371/journal.pone.0094600

**Published:** 2014-04-11

**Authors:** Giacomo Milisenda, Sara Rosa, Veronica L. Fuentes, Ferdinando Boero, Letterio Guglielmo, Jennifer E. Purcell, Stefano Piraino

**Affiliations:** 1 Dipartimento di Scienze e Tecnologie Biologiche ed Ambientali (DiSTeBA), University of Salento, Lecce, Italy; 2 Consorzio Nazionale Interuniversitario per le Scienze del Mare (CoNISMa), Rome, Italy; 3 Department of Animal Biology and Marine Ecology, University of Messina, Messina, Italy; 4 Institut de Ciencies del Mar-CSIC, Barcelona, Spain; 5 Institute of Marine Sciences, National Research Council (ISMAR-CNR), Genoa, Italy; 6 Shannon Point Marine Center, Western Washington University, Anacortes, Washington, United States of America; University of Wales Swansea, United Kingdom

## Abstract

In recent years, jellyfish blooms have attracted considerable scientific interest for their potential impacts on human activities and ecosystem functioning, with much attention paid to jellyfish as predators and to gelatinous biomass as a carbon sink. Other than qualitative data and observations, few studies have quantified direct predation of fish on jellyfish to clarify whether they may represent a seasonally abundant food source. Here we estimate predation frequency by the commercially valuable Mediterranean bogue, *Boops boops* on the mauve stinger jellyfish, *Pelagia noctiluca*, in the Strait of Messina (NE Sicily). A total of 1054 jellyfish were sampled throughout one year to quantify predation by *B. boops* from bite marks on partially eaten jellyfish and energy density of the jellyfish. Predation by *B. boops* in summer was almost twice that in winter, and they selectively fed according to medusa gender and body part. Calorimetric analysis and biochemical composition showed that female jellyfish gonads had significantly higher energy content than male gonads due to more lipids and that gonads had six-fold higher energy content than the somatic tissues due to higher lipid and protein concentrations. Energetically, jellyfish gonads represent a highly rewarding food source, largely available to *B. boops* throughout spring and summer. During the remainder of the year, when gonads were not very evident, fish predation switched towards less-selective foraging on the somatic gelatinous biomass. *P. noctiluca*, the most abundant jellyfish species in the Mediterranean Sea and a key planktonic predator, may represent not only a nuisance for human leisure activities and a source of mortality for fish eggs and larvae, but also an important resource for fish species of commercial value, such as *B. boops*.

## Introduction

In recent years, jellyfish have achieved a prominent position in studies of marine ecology, reflecting their key roles in the pelagic domain; however, their roles in marine food webs may vary according to species, life stages, potential predators, and available resources. Many studies have shown that jellyfish act as predators on both invertebrate (e.g. cnidarians, crustaceans, tunicates) and vertebrate (fish eggs and larvae) zooplankton [1]–[4]. In addition, gelatinous predators may impact trophic webs by impairing the phytoplankton-crustacean-fish pathway [5]–[10].

Due to their high water content, jellyfish are often presumed to be a poor food source and a trophic dead end [11]. Jellyfish were also assumed to be a low-value biomass not readily consumed by higher trophic levels, representing a respiratory sink of carbon, directly leading toward bacterial CO_2_ production [12]; however, in addition to the vertebrate predators that extensively consume gelatinous species, a variety are known to opportunistically/periodically prey on jellyfish [13]. Indeed, the dilute nutritive value of gelatinous organisms could be compensated due to the possibility of rapid digestion and assimilation [14]. Experimentally fed chum salmon, *Oncorhynchus keta*, digested *Pleurobrachia bachei* ctenophores more than 20 times faster than the same wet weight of shrimp and the ctenophores provided adequate nutrition when in sufficient supply to process at this high rate [14]. More recently, *Aurelia aurita* jellyfish proved to be a good additional food when other prey was scarce for the thread-sail filefish, *Stephanolepis cirrhifer* [15].

Because of the rapid digestion, poor preservation, and difficult recognition of gelatinous organisms inside fish digestive systems, fish predation on them has been poorly investigated and probably underestimated [13]. Evidence that jellyfish biomass does not represent a trophic dead end came also from observations of dead biomass of the giant jellyfish *Nemopilema nomurai* sinking to the bottom, where it is consumed by macrobenthic scavengers more rapidly than decomposed by bacteria [16]. Predation on gelatinous plankton can transfer supposed dead-end resources back into the muscle food chain, indirectly favouring an increase in abundance of several piscivorous top predators and affecting the trophic web structure [17]. Recent techniques based on molecular markers (analysis of stable isotopes and fatty acid composition) are rapidly providing information on specialist or opportunistic interactions among gelatinous taxa and their predators [18], [19].

Unfortunately, *in situ* observations on predation on jellyfish are rare [19]–[22]: out of 124 fish species and 34 species of other vertebrates known to use jellyfish as food, most do it occasionally, and just a few are considered as being mainly gelativorous [23], such as leatherback sea turtles, which consume up to 261 jellyfish d^−1^ (330 kg jellyfish wet mass d^−1^) [24]. Some gelatinous plankters also specifically prey on other gelatinous taxa, such as the ctenophore *Beroe ovata* on the sea walnut *Mnemiopsis leidyi* [25]. Intra-guild predation [26] may severely impact jellyfish dynamics, with more than a hundred known predatory interactions among jellyfish taxa [3], [13], [27]–[29]. Jellyfish tissues have lower percentages of carbon than most other zooplankton prey [30], [31], but they may represent a qualitatively important food source for physiological (i.e. growth, reproduction, development) processes [3], [32]. Overall, the small number of predators of jellyfish in any ecosystem has been interpreted that they have a minor impact on jellyfish populations, which instead may be controlled by direct and indirect bottom–up interactions (e.g. crustacean prey availability, primary production) [23]. This view, however, is based on limited information on fish-jellyfish interactions. Field observations of fish predation activity and analysis of species pair interactions can provide new insights on the importance of jellyfish as a trophic resource for fish [33], [34].

In the Mediterranean, *Pelagia noctiluca* (Forsskål, 1775), the mauve stinger, is the most common and conspicuous jellyfish species [35] and at least nine gelativorous fish species have been observed feeding on it (Table 1). Some species of marine turtles, namely *Caretta caretta* and *Dermochelys coriacea*, are also known to include *P. noctiluca* and other jellyfish species in their diets [36], [37] (Table 1). The bogue *Boops boops* (Linnaeus, 1758) is as a gregarious semipelagic fish distributed throughout the Mediterranean Sea and the Black Sea [38]. It lives mainly at depths ≤150 m [38], [39], both near the bottom (especially on rocky and sandy bottoms) and near the surface [40]. It is omnivorous, feeding on both benthic (crustaceans, molluscs, annelids, sipunculids, and plants) and pelagic (siphonophores, eggs, crustaceans, bivalve larvae) prey [41], [42]. Also, *B. boops* has been observed to feed on jellyfish, especially on *P. noctiluca*, but this relationship has never been quantified [28], [43], [44]. Such predatory behaviour of *B. boops* is frequently observed in the Strait of Messina (Sicily), where *P. noctiluca* was first recorded in 1785 [45] and now occurs with regular outbreaks since 1981, with important effects on the planktonic community of the Strait of Messina and the Southern Tyrrhenian and Ionian seas [46].

**Table 1:** Animals reported to feed on *Pelagia noctiluca* in the Mediterranean Sea.

	Predator (*Parasite)	Reference
**Turtles**	Caretta caretta	Bjorndahl, 1997
	Dermochelys Coriacea	Bjorndahl, 1997
**Fishes**	Boops boops	Malej & Vukovic, 1984
	Schedophilus medusophagus	Macpherson & Roel, 1987; Costa, 1991
	Luvarus imperialis	Fitch & Lavenberg, 1968; Costa 1991
	Mola mola	Hart, 1973
	Stromateus fiatola	Haedrich, 1986
	Tetragonurus atlanticus	Haedrich, 1986
	Scomber colias	Relini et al, 2010
	Oblada melanura	Relini et al., 2010
	Tetragonurus cuvieri	Hart, 1973
**Crustacea**	Hyperiid amphipods (*)	Reviewed in Laval, 1980

In this study we investigated for the first time the predation of *B. boops* on *P. noctiluca* in the Strait of Messina (between Italy and Sicily) throughout a year to test whether a) *Boops boops* selectively foraged on its jellyfish prey, and b) selection occurred depending on medusa gender and medusa body part. Qualitative and quantitative characterization of the jellyfish somatic body parts (oral arms, umbrella) and gonads were carried out to elucidate whether the energy content and biochemical composition of jellyfish tissues were related to changes of the observed fish predatory behaviour throughout the year.

## Materials and Methods

### Ethics statement

No specific permits were required for the described field studies in the Strait of Messina. The species collected is the most common native jellyfish in the Mediterranean Sea and is not protected throughout its range. Sampling points (station A: 38° 12' 00 N, 15° 33' 36'' E; station B: 38° 11'43'' N, 15° 35' 58'' E) did not include any protected or private lands.

### Study area

**Figure 1:**
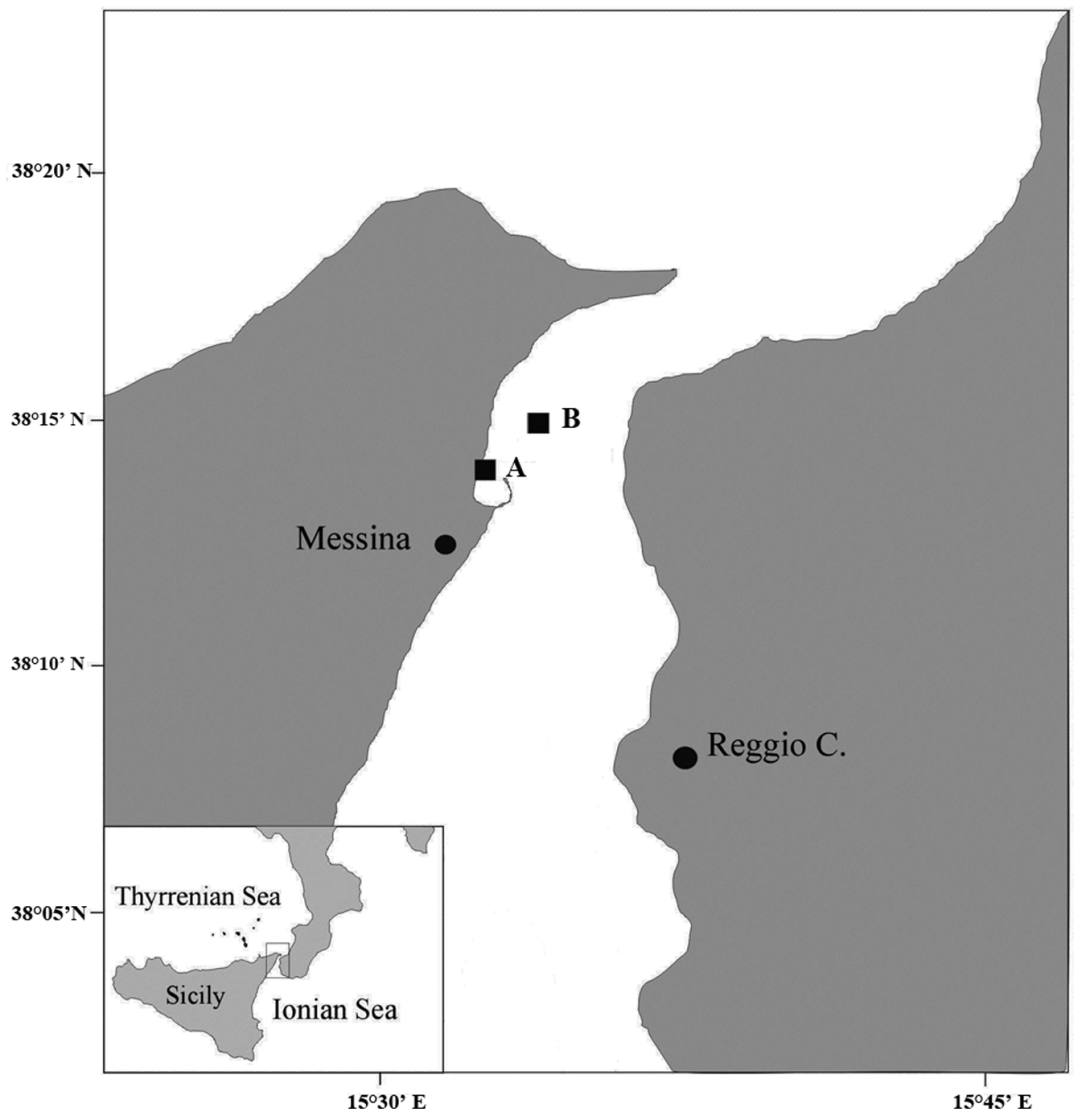
Study area (Strait of Messina). **A** marks the inshore site and **B** marks the offshore site.

The study was carried out in the Strait of Messina, which is geographically located between the Italian peninsula and Sicily, near the Messina Harbour, mainly inshore (Station A in Figure 1). This site is influenced by the peculiar hydrodynamic regime of the Strait, characterized by a six-hour alternation of northward (from the Ionian to Tyrrhenian seas) to southward tidal currents, with upwelling and down-welling water masses reaching up to 200 cm s^−1^ speed [46], which strongly affect the biotic structure and organization of Strait ecosystem. In fact, upwelling systems are one of the most productive marine environments that are characterized by biological richness in all levels of the trophic chain [47]. The hydrodynamic complexity of the Strait ecosystem has a major influence on the horizontal and vertical distribution of the organisms, especially on zooplankton communities. The regular alternation of northern and southern tides, combined with upwelling and downwelling water masses, prevents stratification of the water column. Therefore, the Strait has been compared to an “intermittent pump”, with high inputs of nutrients throughout the autumn and winter seasons, seeding the spring phytoplankton bloom both locally and in adjacent zones [48]–[51].

Due to the peculiar flow of currents and counter-currents, plankton is transported in fronts propagating along the Strait shorelines where, at each current change, *P. noctiluca* jellyfish can reach high surface abundances (up to 8.3 individuals m^−2^; [46]).

### Predation by *Boops boops* on *Pelagia noctiluca*

Underwater videos (Movie S1, Movie S2, Movie S3) to document the predation behaviour of *B. boops* on *P. noctiluca* were recorded at the station A (Figure 1), using a CANON G7 camera within a CANON waterproof case WP DC11 attached to a stick and hand-operated from the surface. Five short videos (5 minutes each) were also recorded in a single day. Each time, the camera was randomly deployed with a fixed frame. All fish-jellyfish interactions recorded in each video were counted by the use of the software VLC media player (http://www.videolan.org/vlc/). The interactions were grouped into four classes based on the number of fish feeding at the same time on an individual jellyfish: 1 - single fish, 2 - low (2–4 fish), 3 - medium (5–10 fish), 4 - high (11–14 fish). The body part eaten (bell, oral arms, and gonads) during each interaction also was reported.

After we observed fish attacks, we conducted a preliminary survey on 20 jellyfish collected inshore to identify specific marks or scars due to *B.boops* predation. These specimens were compared to 20 jellyfish sampled offshore, where no visible *B.boops* schools were preying on jellyfish. The jellyfish collected inshore following fish attacks had bite scars, especially a central hole in the aboral (exumbrellar) or oral (subumbrellar) side of the jellyfish bell that indicated the partial or complete predation of gonads. Fish attacks were also directed towards jellyfish oral arms, which were often partly or sometimes completely eaten in the inshore jellyfish. During our subsequent samplings throughout the year, *B.boops* was the only fish species observed preying on *P. noctiluca*, in spite of the occurrence of several common coastal fish species.

An assessment of the predatory impact of *B. boops* on *P. noctiluca* jellyfish population was made through the analysis of partly devoured jellyfish by observation of missing or damaged body parts (somatic tissue, i.e. bell and oral arms, and gonads; see Movie S1, Movie S2, Movie S3) from jellyfish sampled monthly in the Strait of Messina throughout one year.

**Figure 2:**
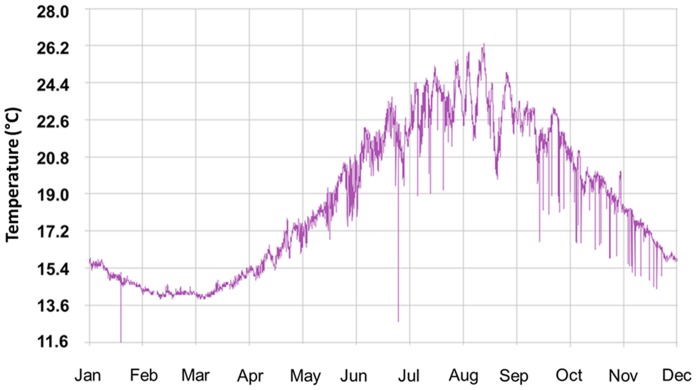
Annual variation of sea surface temperature in the Strait of Messina during 2010. Sea surface temperatures were recorded by the Italian National Tide gauge Network (ISPRA http://www.mareografico.it).

Four seasons were identified based on sea surface temperatures (Figure 2) recorded by the Italian National Tide gauge Network (ISPRA http://www.mareografico.it) through an annual cycle: winter (January–March, 13.6–15.5°C), spring (April–June, 15.4–21.2°C), summer (July–September, 21.2–24.6°C), and autumn (October–December) 20.8–15.4°C). Live *P. noctiluca* specimens were collected from January to December 2010 with a 1-cm mesh size hand net from a boat at the inshore station (station A, Figure 1). To compare the predation frequency between inshore and offshore fish shoals, jellyfish were sampled at an additional location (B) in the Strait of Messina in June 2010 (Figure 1).

**Figure 3:**
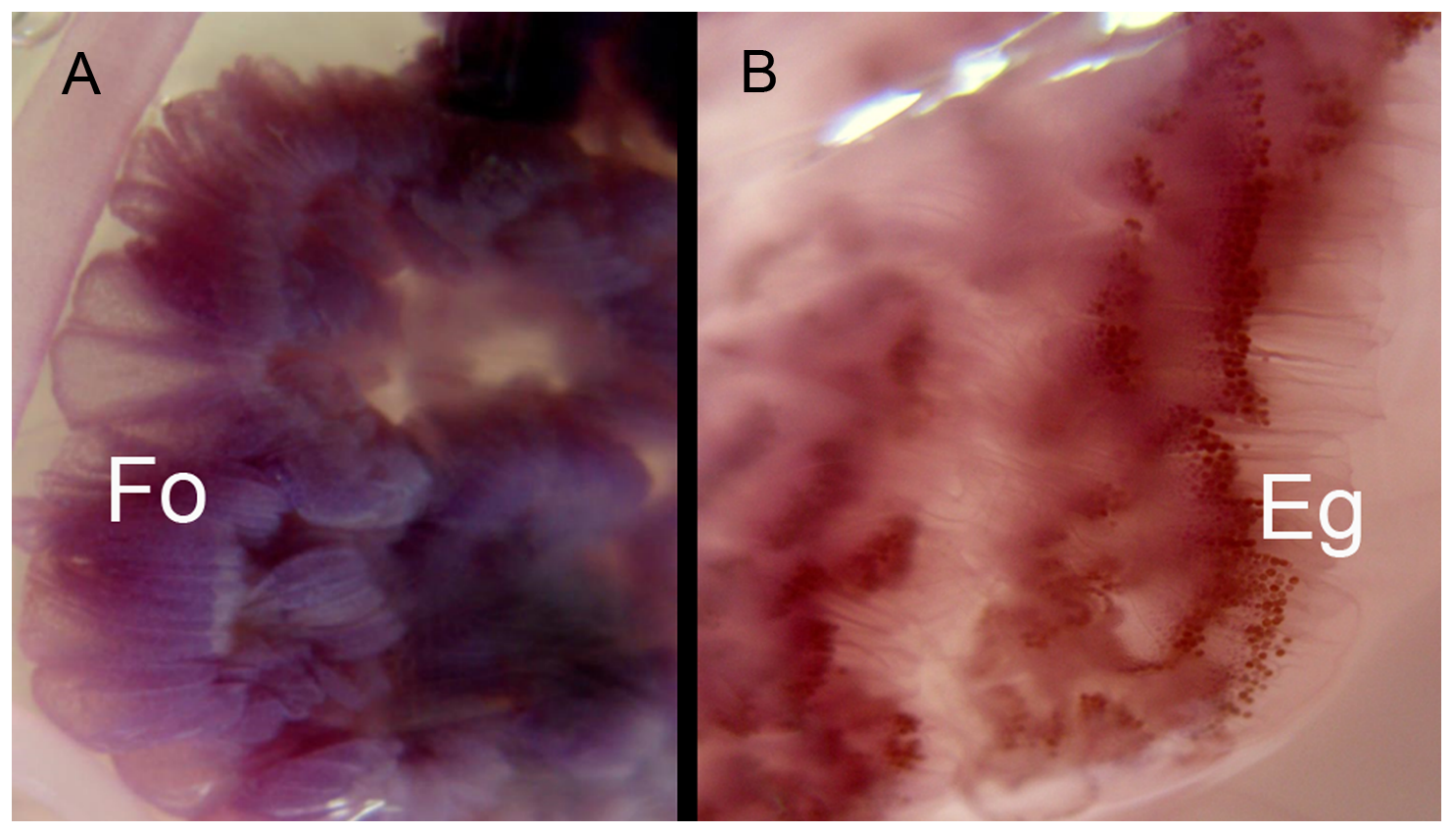
Gonads of *Pelagia noctiluca.* (**A**) male with evident follicles (Fo), (**B**) female with eggs (Eg).

Jellyfish were sampled randomly, in the absence or presence of *B. boops*. On board, jellyfish diameters were measured exumbrellar side up to the nearest millimetre with a calliper and medusa parts consumed by fish were recorded. The gender of all sampled jellyfish was determined by visual analysis of different morphological characteristics of the gonads. Specifically, the male gonad has a dark purple colour and is composed of a series of small cylindrical follicles, stacked together (Figure 3A). The female gonad is pink to red with eggs that can be easily distinguished individually (Figure 3B). For medusae whose gender determination was uncertain visually, a small piece (1 cm) of gonad was removed and stored in 10% formalin for later microscopic analysis. To enable comparisons of unequal jellyfish abundances among different months, the duration of each sampling remained unchanged throughout the year. Absolute data were converted into frequencies of fish predation on different jellyfish genders by season. Data were expressed as mean ± standard errors (SE).

Various jellyfish body parts and tissues were eaten (jellyfish missing oral arms or with damaged umbrella vs. jellyfish with missing gonads). By the observed damage on jellyfish, the Predation Frequency (PF) was calculated either as:

*PF_(m)_*  =  the numbers of male jellyfish damaged (*n_m_*) relative to the number of sampled males (*N_m_*);
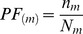

*PF_(f)_*  =  the numbers of female jellyfish damaged (*n_f_*) relative to total number of sampled females (*N_f_*); 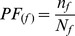

*PF_g_*  =  the numbers of jellyfish with damaged gonads relative to total number of sampled jellyfish;
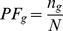

*PF_st_*  =  the numbers of jellyfish with damaged somatic tissue relative to total number of sampled jellyfish;
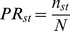
Differences in fish predation were assessed by statistical analysis between jellyfish genders and among different seasons, considering three factors: Season fixed with four levels (Autumn, Spring, Summer, and Winter), Time (triplicate samplings, random and nested in Season, with three levels: t_1_, t_2,_ and t_3_), and Gender, fixed and orthogonal to factors Season and Time, with two levels (Male and Female). Homogeneity of variances was tested by Cochran's C test. Data were analysed using 3-way permutational multivariate analysis of variance (PERMANOVA) [52].

Second, we tested for differences in predation among different body parts of the jellyfish, among the genders and seasons, considering an additional fourth factor, Body Part, fixed and orthogonal to factors Season, Time, and Gender, with two levels (Somatic Tissue and Gonads). Data were analysed using 4-way PERMANOVA [52], after ensuring homogeneity of variances by means of Cochran's C tests.

During the Spring period, we compared the fish predation on medusae inside and outside the Messina Harbour (Figure 1). The test considered two factors: Location, fixed with two levels (inside and outside the harbour), and Site, random and nested in Location, with three different levels (S_1_, S_2,_ and S_3_). Thirty jellyfish were analysed for each site, totalling 180 *P*. *noctiluca* medusae. Data were analysed using 2-way PERMANOVA [52], after ensuring homogeneity of variances by means of Cochran's C tests. A chi-square test was used to test whether the sex ratios were significantly different from the expected ratio of 1∶1.

### Energy content of medusa tissues

Each jellyfish was dissected to isolate gonads and oral arms, which were immediately rinsed in distilled water to remove salt, wiped with blotting paper, and weighed with an electronic 1 mg precision balance. The swimming bells were not included in the energy content analysis, because the majority of predation events occurred on gonads and oral arms only. These body parts were shown to possess the highest energy density in other jellyfish [53], more than five times higher in the gonads than in the bells. Samples were dried in an oven at 60°C to constant weight [54] and the energy density of dry mass (DM) was determined for each body part using a Phillipson micro bomb calorimeter and expressed as J mg DW^−1^. Calorimetric values for each part (gonads and oral arms) were calculated as in Doyle et al. (2007) [53]. The statistical design considered two factors: Gender, fixed with two levels (Male and Female); and Body Part, fixed and orthogonal in Gender, with two levels (Oral arms and Gonad). Homogeneity of variances was tested by Cochran's C test. Data were analysed using 2-way PERMANOVA and visualized by a non-Parametric Multi-Dimensional Scaling (nMDS) ordination model [52]. In this case, 60 jellyfish were analysed for each gender, totalling 120 specimens of *P. noctiluca*.

### Biochemical analyses

Biochemical analyses to determine the organic matter (OM) composition in carbohydrates, proteins, and lipids were carried out using 20 medusae. Gonadal and somatic tissues were frozen in liquid nitrogen, temporarily stored at −20°C, and transferred at −80°C one hour before lyophilisation to facilitate the freeze-drying process (48 h). Quantification of carbohydrates, proteins and lipids was carried out by colorimetric determination at 480 nm, 750 nm, and 520 nm, respectively. For carbohydrates determination, approximately 7 mg (±0.1 mg) of each lyophilized tissue sample was homogenized in 3 ml of double distilled water [55], with glucose as a standard. For proteins, approximately 7 mg (±0.1 mg) of each lyophilized tissue sample was homogenized in 2 ml of 1N NaOH [56], with albumin as a standard. Finally, approximately 10 mg (±0.1 mg) of each lyophilized tissue sample was homogenized in 3 ml of chloroform-methanol (2∶1) for total lipid determination [57], with cholesterol as a standard. Quantities were expressed as µg mg^−1^ of OM.

To detect differences in biochemical composition and in the content of organic and inorganic matter between gonads and somatic tissue, data were analysed using one-way PERMANOVA, after ensuring homogeneity of variances by means of Cochran's C tests. Differences were further investigated by means of the SIMPER method to highlight the biochemical component(s) contributing most to such differences [52].

## Results

### Predation behaviour of *Boops boops*

Video recordings showed moderate to intense fish-jellyfish interactions, up to dense fish aggregations feeding on the same jellyfish (Table 2; Movie S1, Movie S2, Movie S3). As a result, jellyfish were entirely or partly devoured. Conversely, other abundant near-shore fish species, such as *Chromis chromis*, were not observed to feed on jellyfish (Movie S1).

**Table 2:** Predatory behaviour of *Boops boops* feeding on *Pelagia noctiluca* from *in situ* video analysis.

Fish aggregation level	Number of events	Parts predated
**Single fish**	16	Oral arms
**Low (2–3 fish)**	7	Oral arms
**Medium (5–10 fish)**	10	Oral arms/Gonads
**High (11–14 fish)**	4	Gonads

During 25 minutes of video recordings, 37 distinct predation events were quantified *in situ*. In term of duration of predatory events, the contact between *B. boops* and *P. noctiluca* represented 56% of the total recorded videos, equal to 14 minutes. The contacts of the longest duration occurred when several fish were feeding simultaneously on the same jellyfish prey, while predatory events on single fish lasted only a few seconds. We distinguished three patterns of fish aggregations, depending on the number of fish and the body part of the jellyfish eaten (Table 2). Single fish were always consuming jellyfish oral arms and such individual feeding did not result in additional fish arriving. Conversely, groups of 6–10 fish were associated with ingestion of both jellyfish oral arms and gonads (Movie S1, Movie S2, Movie S3), whereas groups of 13–14 fish were observed feeding only on gonads from a single jellyfish. Usually, many fish aggregated when jellyfish gonads were exposed as a result of repeated bites by single fish attacks.

### Predation by *Boops boops* on *Pelagia noctiluca*

**Figure 4:**
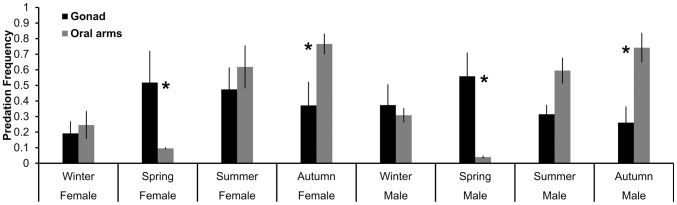
Seasonal Predation Frequency (mean ± SE) of *Boops boops* according to gender and body part of *Pelagia noctiluca*. Asterisks (*) mark significant differences at *p*≤0.05.

From January to December 2010 a total of 1054 jellyfish were sampled, 583 males and 471 females. Overall fish predation (Figure 4) differed among seasons at the 0.1 level (F_0.05, 3, 8_ = 3.41; P = 0.08) and pairwise differences were significant at the 0.05 level between winter (0.45±0.13) and summer (0.88±0.06) (P = 0.033, df = 1). Fish predation did not differ between male and female jellyfish either seasonally (Figure 4) or throughout the year (Female: 0.67±0.09; Male: 0.71±0.11); however, the PERMANOVA analysis showed significant interactions in Gender x Season (F_0.05, 3, 8_ = 6.16; P = 0.018) and the pair-wise tests showed significant interactions only for females between seasons: Winter vs. Summer (0.34±0.16 vs. 0.91±0.1; P = 0.007) and Winter vs. Autumn (0.34±0.16 vs. 0.86±0.12; P = 0.012, df = 1). The numbers of male *P. noctiluca* generally were higher than females and the differences were statistically significant in September and over the year (Table 3).

**Table 3:** Chi-square analyses to test for significant differences in gender distribution of *Pelagia noctiluca*.

	Male	Female	Sum	Expected Values	X^2^
**January**	76	72	148	74	0.05
**February**	38	25	63	31.5	1.34
**March**	18	5	23	11.5	3.67
**April**	55	38	93	46.5	1.55
**May**	34	22	56	28	1.29
**June**	28	11	39	19.5	3.71
**July**	31	30	61	30.5	0.01
**August**	40	36	76	38	0.11
**September**	56	20	76	38	* 8.52
**October**	61	61	122	61	0.00
**November**	75	84	159	79.5	0.25
**December**	71	67	138	69	0.06
**Annual**	583	471	1054	527	* 5.95

(X^2^_(0.05)_ = 3.84). Asterisk (*) marks significant difference.

Predation differed between body parts (somatic tissue vs. gonads), by jellyfish gender, and season (Figure 4). PERMANOVA analysis showed significant differences in the interactions Season x Body Part (F_0.05, 3, 8_ = 7.44; P = 0.01) and Gender x Body Part (F_0.05, 3, 8_ = 14.08; P = 0.0001, df = 1). Predation differed significantly between gonads and somatic tissue for both sexes only in Spring (P = 0.002, df = 1) and Autumn (P = 0.002, df = 1).

Finally, fish predation differed significantly between the two locations (F_0.05, 1, 98_ = 46.27; P = 0.0001, df = 1), with higher predation in the littoral harbour area (0.83±0.06) than offshore in the central channel of the Messina Strait (0.30±0.04). Predation at the inshore sampling stations was 2.7 times higher than at the offshore sampling station.

### Energy quantification and biochemical analysis

**Figure 5:**
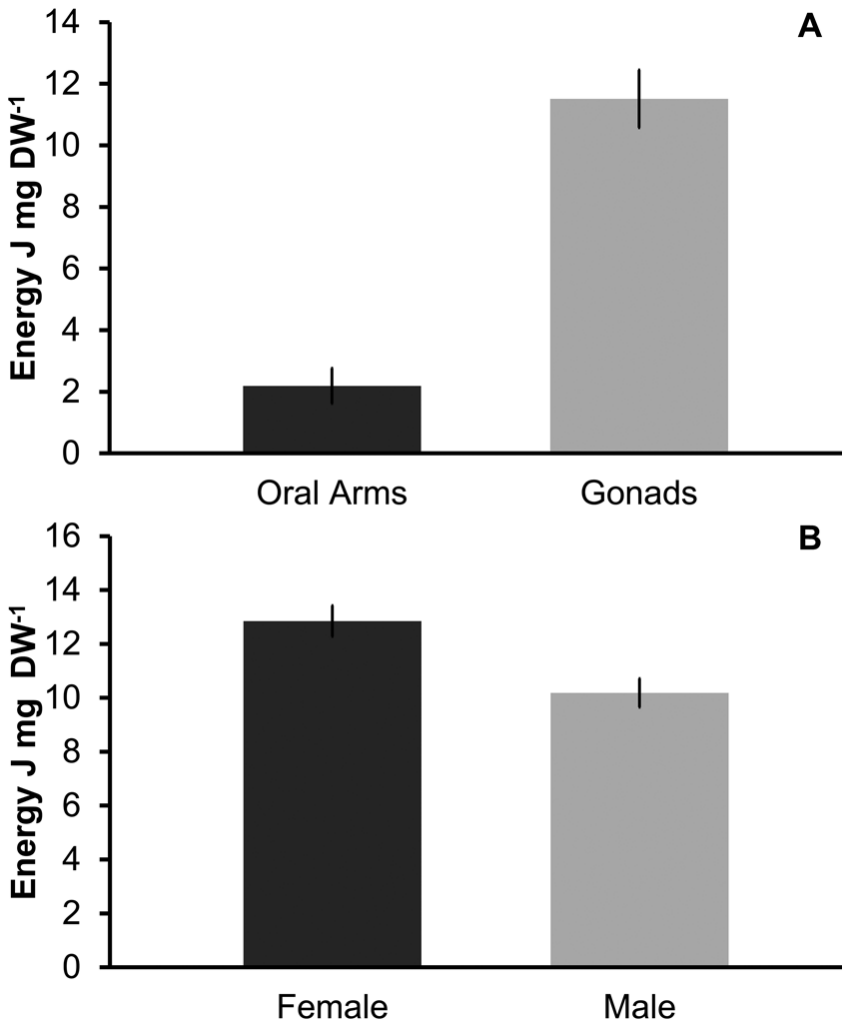
(A) Energy content of the oral arms and gonads of *Pelagia noctiluca*. (B) Energy content of the female and male gonadal tissue of *Pelagia noctiluca*.

**Figure 6:**
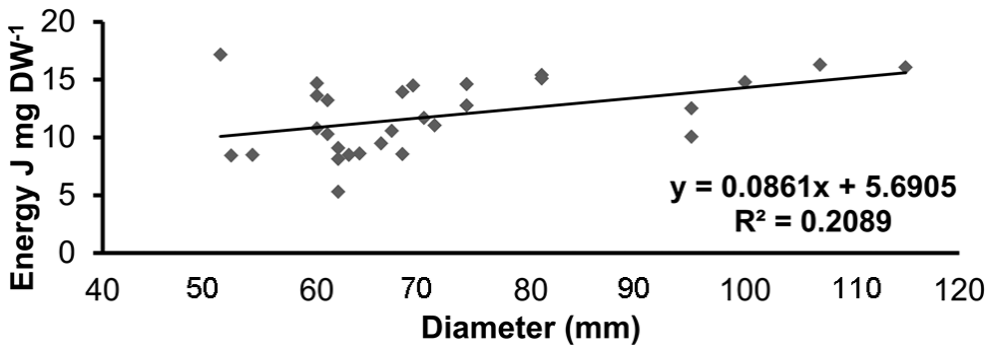
Linear regression between umbrella diameter and gonadal energy content of female *Pelagia noctiluca*.

The energy content of jellyfish differed significantly between the gonads (11.51 J mg DW^−1^) and somatic tissue (2.19 J mg DW^−1^) (F_0.05, 1, 23_ = 12.85; P = 0.003), emphasizing the high energetic value of the gonadal tissue (Figure 5A). Female gonads (12.85 J mg DW^−1^) were significantly (F_0.05, 1, 45_ = 10.12; P = 0.003) enriched energetically compared to male gonads (10.18 J mg DW^−1^) (Figure 5B), whereas no differences were detected between male and female somatic tissues. Furthermore, the energetic content of the female gonads was positively correlated with jellyfish size (P = 0.012), reflecting increasing gonad maturation in larger individuals (Figure 6).

**Figure 7:**
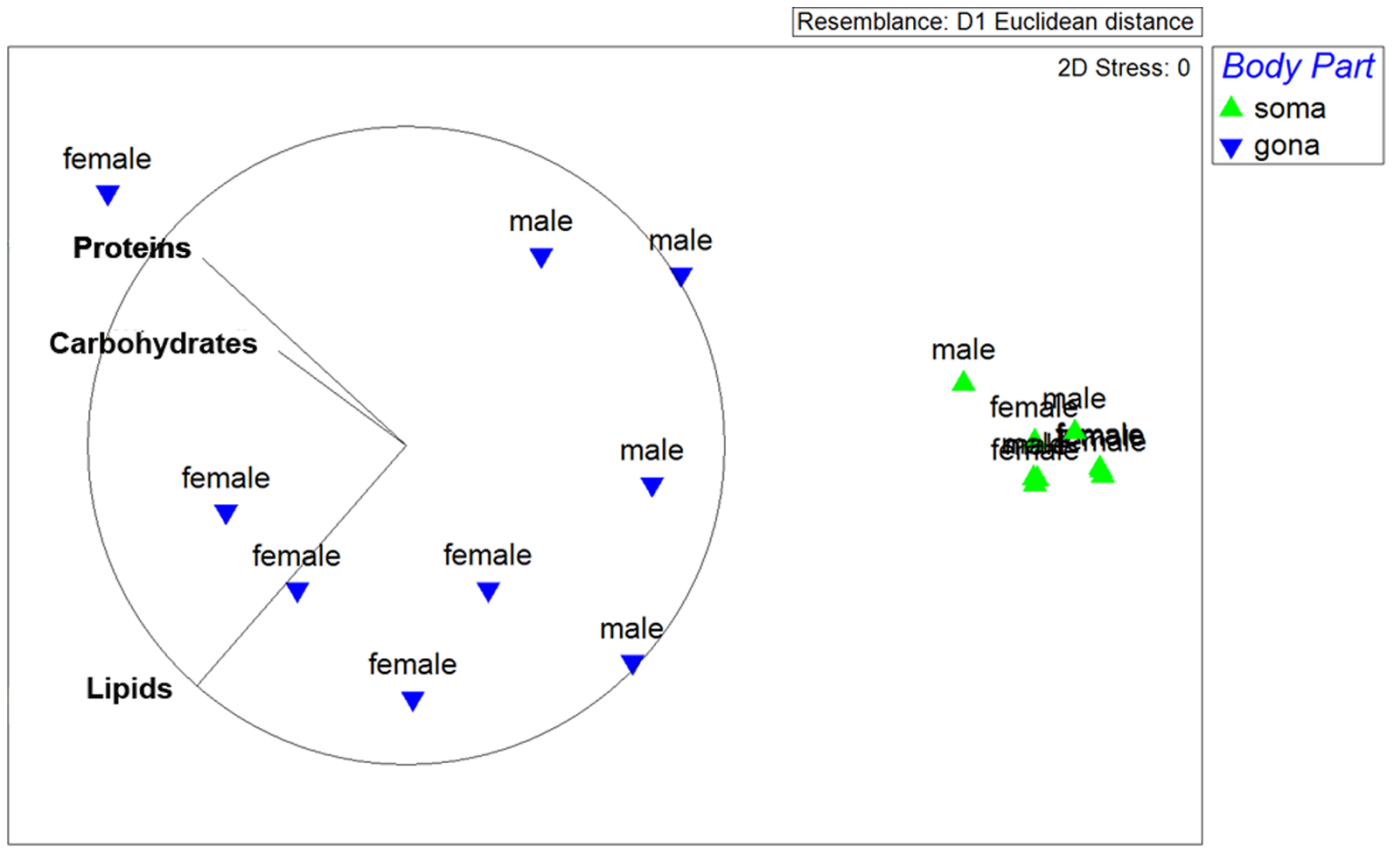
Biochemical composition of *Pelagia noctiluca* (protein, carbohydrate and lipid concentrations): non-Parametric Multi-Dimensional Scaling ordination model for the combined factors Gender x Body Part. Pearson's correlation for each macromolecular group along MDS1 and MDS2 axes is plotted as vectors. (soma) somatic tissue, (gona) gonadal tissue.

**Figure 8:**
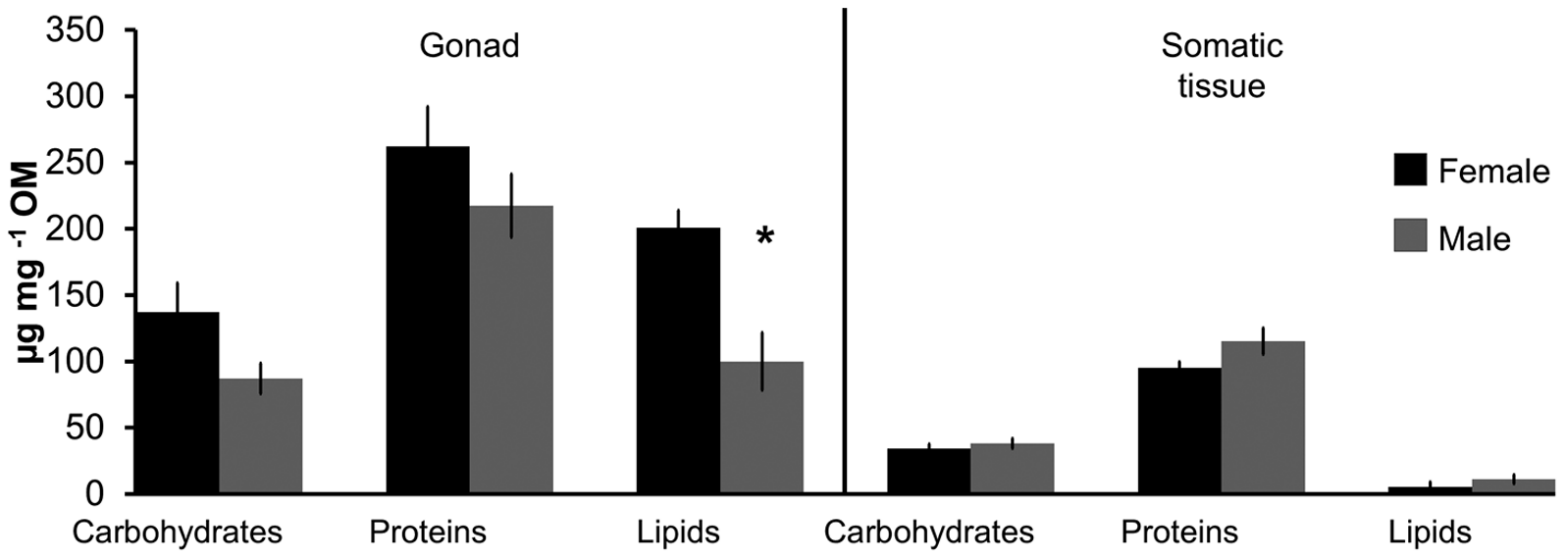
Amount of biochemical components according to different body parts and gender of *Pelagia noctiluca*. OM = organic matter. Asterisk (*) marks significant difference at *p*≤0.05

The biochemical composition differed markedly between the somatic and gonadal tissues of *P. noctiluca* (F_0.05, 1, 14_ = 97.85; P = 0.001) and also between genders (F_0.05, 1, 14_ = 3.05; P = 0.02), as detected by the PERMANOVA analysis. The nMDS ordination showed a clear separation between somatic tissue and gonadal tissue in both genders. Furthermore, the model highlights the higher homogeneity of biochemical composition of the somatic tissues between genders respect to the gonadic tissues (Figure 7). Person's correlation of biochemical compounds along the two axis (MDS1 and MDS2) are plotted as vectors, whose lengths and orientations show that lipids and proteins contributed most to the heterogeneity of samples. The pair-wise tests showed significant differences in the interaction between gender only for gonadal tissue (P = 0.036, df = 1). The SIMPER analysis indicated that most of the difference between the gonadal and somatic tissues was due to the total lipid (41%) and protein contents (38%). Proteins, lipids, and carbohydrates were differently distributed between gonads and somatic tissues (F_0.05, 1, 16_ = 43.60, P_proteins_ = 0.0001; F_0.05, 1, 16_ = 43.12, P_lipids_ = 0.0004; F_0.05, 1, 16_ = 26.15, P_carbohydrates_ = 0.001), both in males and females (Figure 8), with higher concentrations in the gonads, but gender differences were significant only for lipid contents of gonadal tissue (P = 0.036, df = 1).

**Figure 9:**
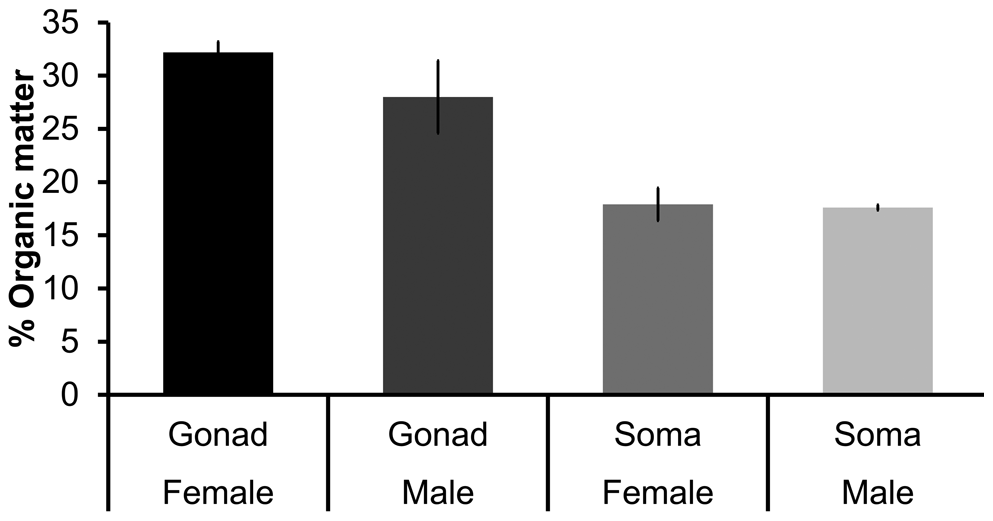
Percentage of organic matter according to different body parts and gender of *Pelagia noctiluca*.

The amount of OM differed (F_0.05, 1, 14_ = 38.89; P = 0.001) between the two tissues (Figure 9), with the gonads containing a higher percentage of OM than the somatic tissue. Male and female jellyfish showed no significant differences composition of organic and inorganic matter in both the gonadal and somatic tissues.

## Discussion

To our knowledge, this is the first assessment of the trophic interaction between a fish and its jellyfish prey throughout an annual cycle. Our analysis documented that *B. boops* foraged on jellyfish all year long, increasing from winter to summer months and following the sinusoidal pattern of the sea surface temperature (Figure 2). This pattern may be explained by the observed spring recruitment of new cohorts of *B. boops* in the Mediterranean Sea [58], [59]. By contrast, *P. noctiluca* abundance in the Strait of Messina peaks in spring and declines in summer months [46]. The occurrence of alternative zooplankton prey (200–500 µm) was highest in early spring (May, 0.85 mg DM m^−3^) and late summer (August, 1.5 mg DM m^−3^) (Milisenda, unpublished data), maximizing food availability for omnivorous bentho-pelagic predators like bogues. Therefore, the increasing *B. boops* predation on jellyfish in spring and summer cannot be explained by the lack of alternative food sources.

The analysis of fish foraging behaviour on jellyfish revealed that inter-seasonal differences in predation were due to a significant variation of predation on female jellyfish only (Figure 4). This can be related to the seasonal development of oocytes and the bimodal onset of the vitellogenic processes in *P. noctiluca* [60]. This coincides with the increase of *B. boops* predation on female *P. noctiluca* specimens, as well as with the summer maximal abundance of mature oocytes, bearing increasing proportions of energy-rich, lipoprotein-containing vesicles in the egg yolk [60]. The highest energy content of gonadal to somatic tissue as well as the jellyfish size-dependent energy content of gonads is clearly demonstrated by our calorimetric analysis, which quantified a six-fold energetic difference between the gonads and the oral arms of *P. noctiluca* and significantly higher energy content of female gonads (Figures 5 and 6). Conversely, the lowest frequency of predation was observed in wintertime (Figure 4), coincident with reduced proportions of mature oocytes in *P. noctiluca* [60]. In this period *P. noctiluca* abundance is still high [46] but the frequency of predation is lower than in warmer months. This suggests that the exploitation of jellyfish as prey is not mainly governed by its availability.

The production of water-soluble molecules related to gonad maturation might act as a cue for *B. boops* predation. Gametogenesis, in fact, is controlled by an endogenous neuro-hormonal induction of maturation and shedding and by environmental (i.e. biological, chemical, and physical) controls modulating reproduction [61]. When jellyfish were attacked for gonads, which become exposed to predators through holes in the upper exumbrellar surface, *B. boops* exhibited mob foraging (Movie S1, Movie S2, Movie S3) by large groups. Specific experiments are needed to identify sensory mechanisms (olfactory, gustatory, visual) driving fish aggregation on jellyfish [62].

Predation frequency was much higher inshore than offshore, due to the strong preference of *B. boops* to shoal along inshore waters on sand, rocks, *Posidonia oceanica* (phanerogamous seagrass) meadows [40], and also near artificial seawalls [63]. Furthermore, the inshore location (station A) is subjected daily to higher concentrations of jellyfish than in deeper waters, due to the alternation of tidal currents flowing across the Strait of Messina. These currents are known to produce gyres along the coastal areas, leading to the patchy accumulation of planktonic organisms [46]. Nevertheless, our analyses were limited to surface collections and predation activity in deeper waters is unknown.

In addition to prey availability, selective foraging behaviour of predators is crucial to understand species pair (predator – prey) interaction strength in food webs. Indeed, optimisation of foraging activities would result in maximizing growth, reproductive potential, and eventually fitness of the predator species [64]. Previous examples exist for selective feeding of both vertebrate and invertebrate predators on gonads [65], [66]. The energy content of the gonads of *Chrysaora hysoscella*, *Rhizostoma octopus*, *Cyanea capillata* were much higher than other tissues, suggesting that these differences may influence foraging decisions of turtles feeding on jellyfish [53]. A preferential use of prey regions is probably widespread. Brown bears (*Ursus arctos*), for example, prey on salmon (*Oncorhynchus nerka*) in a seasonal pattern, displaying partial and selective consumption depending on the relative availability and attributes of the fish [67]. When salmon availability is high, bears target mainly energy-rich body parts (i.e. gonads and brain). During periods of low salmon abundance, bears switch to a less-selective consumption of their prey. Comparably, resident killer whales (*Orcinus orca*) in British Columbia for most of the year feed selectively on the largest salmon (*Oncorhynchus tshawytscha*) species with the highest lipid content at rates far exceeding their relative seasonal availability compared to alternative prey [68]. The selective foraging behaviour of *B. boops* during spring months (Figure 4) may be due to the higher availability of large specimens of *P. noctiluca* (15–20 cm) [46], when the jellyfish produced gonads with more OM, energetic value, and lipids than somatic tissues. Female gonads, because of the oocyte maturation and yolk storage, were the most valuable food item throughout spring and summer. Consequently, the observed *P. noctiluca* sex ratio might be influenced by the slight preference observed in the foraging behaviour of *B. boops* for the female jellyfish gonads.

Enclosure experiments showed that roach (*Rutilus rutilus*) predation on the copepod *Eudiaptomus gracilis* may significantly reduce the numbers of the reproductive female copepods [69] due to their high visibility. Similarly, the presence of gonads in *P. noctiluca* may increase their visibility to foraging fish, eliciting higher predatory pressure on their purple-red pigmented gonads (Figure 3) than on their translucent somatic tissue. Spawning and fish predation on jellyfish gonads would progressively reduce this resource, causing *B. boops* to switch towards a less-selective foraging behaviour and exploit the abundant gelatinous somatic biomass of *P. noctiluca*.

Jellyfish gonad ripeness is positively correlated with the occurrence of parasitoid hyperiid amphipods [70] and a number of species are reported to be associated with *P. noctiluca* [71]. Several fish are known to feed on those amphipods [13], [72], however, among the 1054 sampled *P. noctiluca*, we never observed any associated hyperiid amphipods. Therefore, the foraging behaviour of *B. boops* did not appear to be related to the presence of associated animals.

Our results demonstrate that *P. noctiluca* can represent an important food source for gelativorous predators both by its increased energy content during the period of gonad maturation [54] and by the high available biomass during spring and summer outbreaks [46]. Fish predation may also affect *P. noctiluca* populations and their dynamics through reduced reproduction due to predation on the gonads and reduced feeding and growth due to predation on the oral arms and gastric pouches with the gonads. Quantification of such predation could lead to a better understanding the mechanisms and dynamics of jellyfish blooms. Also, fish-jellyfish species pair interactions provide new information for fishery management in coastal waters, which could take advantage of recurrent jellyfish blooms to maximize seasonal yields of jellyfish-eating fish species, or conversely, by protection or enhancement of effective jellyfish predators as countermeasures against problematic jellyfish.

## Supporting Information

Movie S1 ***Boops boops*** **fish aggregation feeding on** ***Pelagia noctiluca.***
(WMV)

Movie S2 ***Boops boops*** **feeding on oral arms of** ***Pelagia noctiluca.***
(WMV)

Movie S3 **Movie on** ***Boops boops*** **feeding on gonads of** ***Pelagia noctiluca.***
(WMV)
